# *KRAS* wild-type pancreatic ductal adenocarcinoma: molecular pathology and therapeutic opportunities

**DOI:** 10.1186/s13046-020-01732-6

**Published:** 2020-10-28

**Authors:** Claudio Luchini, Gaetano Paolino, Paola Mattiolo, Maria L. Piredda, Alessandro Cavaliere, Marina Gaule, Davide Melisi, Roberto Salvia, Giuseppe Malleo, Jae Il Shin, Sarah Cargnin, Salvatore Terrazzino, Rita T. Lawlor, Michele Milella, Aldo Scarpa

**Affiliations:** 1 Department of Diagnostics and Public Health, Section of Pathology, University of Verona, 37134 Verona, Italy; 2 ARC-Net Research Center, University and Hospital Trust of Verona, 37134 Verona, Italy; 3 Section of Oncology, Department of Medicine, University and Hospital Trust of Verona, Piazzale L.A. Scuro 10, 37134 Verona, VR Italy; 4 Department of Surgery, University of Verona, 37134 Verona, Italy; 5 Yonsei University College of Medicine, 03722 Seoul, Republic of Korea; 6 Department of Pharmaceutical Sciences and Interdepartmental Research Center of Pharmacogenetics and Pharmacogenomics (CRIFF), University of Piemonte Orientale, 28100 Novara, Italy

**Keywords:** *KRAS*, *BRAF*, MSI, dMMR, fusion genes, pancreatic cancer

## Abstract

Pancreatic ductal adenocarcinoma (PDAC) is a deadly disease, whose main molecular trait is the MAPK pathway activation due to *KRAS* mutation, which is present in 90% of cases.

The genetic landscape of *KRAS* wild type PDAC can be divided into three categories. The first is represented by tumors with an activated MAPK pathway due to *BRAF* mutation that occur in up to 4% of cases. The second includes tumors with microsatellite instability (MSI) due to defective DNA mismatch repair (dMMR), which occurs in about 2% of cases, also featuring a high tumor mutational burden. The third category is represented by tumors with kinase fusion genes, which marks about 4% of cases. While therapeutic molecular targeting of *KRAS* is an unresolved challenge, *KRAS*-wild type PDACs have potential options for tailored treatments, including *BRAF* antagonists and MAPK inhibitors for the first group, immunotherapy with anti-PD-1/PD-L1 agents for the MSI/dMMR group, and kinase inhibitors for the third group.

This calls for a complementation of the histological diagnosis of PDAC with a routine determination of *KRAS* followed by a comprehensive molecular profiling of *KRAS*-negative cases.

## Background

Pancreatic ductal adenocarcinoma (PDAC) is the seventh leading cause of global cancer-related deaths in industrialized countries, projected to become the second most common in the next decade worldwide [1–3]. More than 80% of PDAC patients present with locally advanced or metastatic disease, not amenable to surgical resection with curative intent, at the time of diagnosis [3, 4]. Despite progress in the chemotherapeutic treatment of PDAC, long-term survival remains dismal, with less than 10% of patients alive 5 years after diagnosis [5, 6]. To improve PDAC survival significantly, new therapeutic strategies are needed. One of the most promising tools is represented by the integration of histology with molecular pathology, to identify potential molecular targets for tailored treatments [7].

From a genetic point of view, PDAC is a composite disease, showing a very complex network of point mutations, epigenetic alterations and chromosomal structural variants [8, 9]. Within this complexity, however, the master driver is the *KRAS* (Kirsten rat sarcoma) oncogene [8–10], mutationally activated in over 90% of cases and recently reported to be more common in older (≥50 years) and female patients [11]. Notably, KRAS mutations can be detected with reliable sensitivity and specificity also through liquid biopsy in PDAC patients [12].

KRAS encodes a small GTPase, which acts as a transducer-effector, cooperating with cell surface receptor tyrosine kinases [10]. Once triggered, it activates different intracellular pathways involved in carcinogenesis, such as proliferation and cell migration, evasion of the immune system, and block of apoptosis [10]. Among the different pathways interrelating with KRAS function, the mitogen-activated protein kinase (MAPK) pathway is a crucial one [8–10].

Although many therapeutic efforts have been made to target the function of mutated KRAS oncoprotein, they have been unsuccessful in substantially modifying PDAC prognosis. For example, three recent phase 2 trials evaluated MAPK inhibitors alone or in combination with gemcitabine in locally advanced and/or metastatic PDAC, and failed to show any survival benefit [13–16]. Other efforts include 4 studies that evaluated KRAS vaccines in PDAC patients: three studies that looked at RAS peptide vaccines and one that used GI-4000, a tarmogen (targeted molecular immunogen) designed to target cells with mutant *KRAS* [17–21]. Furthermore, the targeted drug tipifarnib that inhibits the Ras-dependent growth of cancer cells was tested in a large phase 3 trial and a phase 1 dose escalation trial in combination with gemcitabine in advanced PDAC [22, 23]. However, none of these studies achieved significant survival benefits in PDAC patients. Notably, a recent report regarding extracellular signal-regulated kinases (ERK) in *KRAS*-mutated PDAC, based on cell lines, pancreatic cancer organoids, and xenograft mouse-models, showed that ERK inhibition in cancer-associated stromal cells can suppress cancer-stromal interactions and PDAC metastatic potential [24]. Along these lines, Sun and colleagues, reported that inhibition of the phosphorylation of transgelin-2, a novel target of KRAS-ERK signaling, may be a potential therapeutic strategy for targeting PDAC with *KRAS* mutation, using PDAC cell lines, immunohistochemistry on PDAC tissues, and xenograft-mouse models [25]. Although promising, such findings are exploratory and still without a direct clinical translation. These points highlight the difficulties in targeting *KRAS*, and this situation still represents one of the most important reasons that can explain the high-mortality rate of PDAC also in the era of precision oncology [10, 26]. Moreover, Kim and colleagues reported that PDAC patients with *KRAS* mutations display a worse response to gemcitabine-based chemotherapy and shorter overall survival than those with *KRAS* wild-type [27]. Along the same lines, Windon and colleagues confirmed a survival disadvantage for *KRAS* mutant patients, showing that such adverse prognostic effect was independent of mismatch repair status and the specific chemotherapy regimen employed [28]. These findings warrant further investigation, as they may support new strategies for implementing precision oncology in PDAC patients. For example, using a specific gene signature for KRAS dependency, recently led to the identification and validation of decitabine as a potent inhibitor of growth in KRAS-dependent pancreatic cancer cells and patient-derived xenograft models [29], an approach that is now being translated to the clinic.

Intriguingly, a not-negligible proportion of PDAC (about 8–12%) does not harbor *KRAS* mutations [3, 8, 9, 11]. The definition of the genetic landscape of *KRAS* wild-type PDAC has been recently improved by whole-genome sequencing studies [8, 11], which highlighted the occurrence of several genetic alterations representing potential targets for tailored therapy. At this time, *KRAS* wild-type tumors are the molecular PDAC subgroup that might receive the highest prognostic benefits from precision oncology.

## Molecular pathology and therapeutic opportunities

**Fig. 1:**
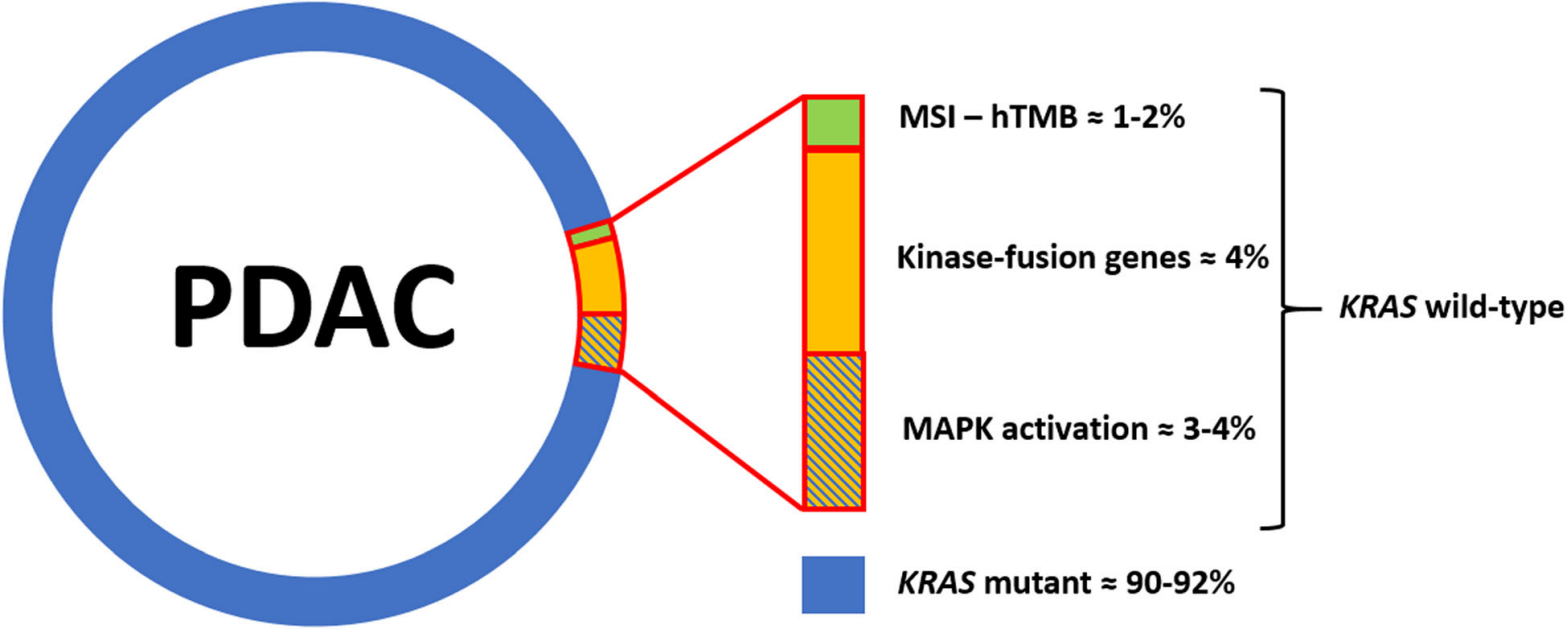
The genetic landscape of *KRAS* wild-type pancreatic ductal adenocarcinoma (PDAC) is shown. The vast majority of cases harbored *KRAS* mutations, but about 8–10% of cases show other molecular alterations, including microsatellite instability (MSI) – high tumor mutational burden (hTMB), kinase fusion genes, and activation of the MAPK pathway without *KRAS* involvement

The genetic landscape of *KRAS*-wild type PDAC can be subdivided into three different groups: i) PDAC with the presence of an activated-MAPK in the absence of a *KRAS* mutation, ii) PDAC with microsatellite instability/defective DNA mismatch repair, and iii) PDAC with kinase-fusion genes. These alterations dominate the *KRAS* wild type PDAC genetic landscape, and occur in different proportions (Fig. 1). This review will focus on molecular pathology and therapeutic opportunities in these specific PDAC molecular settings.

### The MAPK pathway and the role of *BRAF* (B-Raf proto-oncogene)

The MAPK pathway is the most important core signaling pathway in PDAC [3, 8, 9, 11, 26], in which the activation of RAS is a crucial step. Such activation is mediated by a guanine nucleotide exchange factor [30]. Active RAS is self-inactivated by intrinsic GTPase activity, based on a GTPase-activating protein [31]. *KRAS* mutations in PDAC often involve codons 12, 13 and 61; most of them are G12D or G12R substitutions, which decrease the intrinsic GTPase activity, thus resulting in prolonged activation of RAS [31].

Notably, the activation of MAPK pathway is possible also in the absence of *KRAS* mutations [30]. A recent study by Singhi et al. definitively demonstrated that the MAPK signaling is activated in about one third of *KRAS* wild-type PDAC [11]. In this cohort, *BRAF* alterations were the most prevalent, and included activating missense mutations, amplification and kinase fusions. *BRAF* mutations were mutually exclusive with those of *KRAS*; furthermore, kinase fusions in *BRAF* were not present in *KRAS* mutant cases [11]. *BRAF* encodes for the B-RAF proto-oncogene, a serine/threonine kinase belonging to a family of MAPK kinases [30]. The most common mutations in BRAF usually involve codon 600 and often results in a V600E substitution, leading to constitutive activation of its kinase function, which is the results also of the other aforementioned alterations [32, 33]. The mutual exclusivity of *KRAS* and *BRAF* mutations in PDAC suggests that the activating mutations of these genes can compensate for each other in PDAC oncogenesis via activation of the MAPK pathway [30]. Recent data also suggest that other molecular alterations may modulate cancer cell (including PDAC) susceptibility to MAPK inhibition: indeed, *PTEN* loss was shown to portend intrinsic resistance to MEK inhibitors and synergistic activity of combined MAPK/phosphatidylinositol 3 kinase (PI3K) pathway inhibition [34]; however, combined MAPK/PI3K inhibition did not prove effective in either cell line models of PDAC [35] or PDAC patients [36], presumably owing to the lack of *PTEN* alterations.

**Fig. 2:**
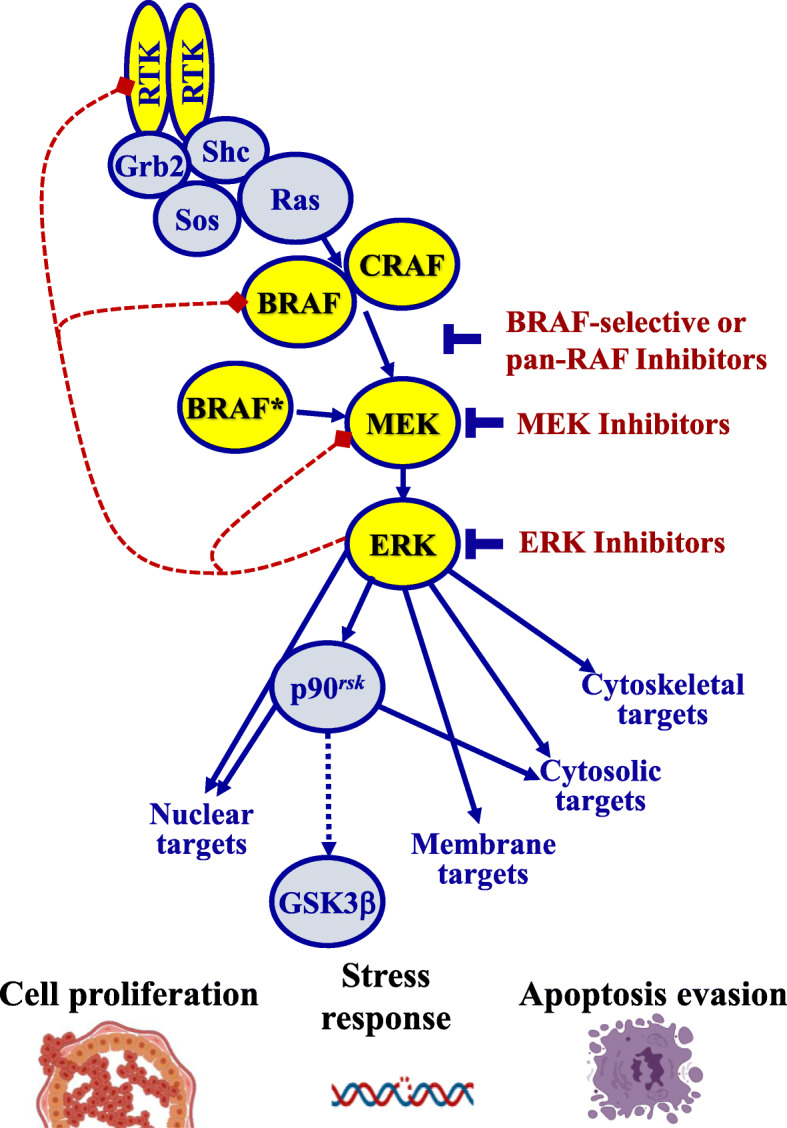
A schematic representation of the MAPK pathway is here shown. As highlighted, B-RAF is an immediately downstream of RAS and triggers MAP 2 K1/MEK, which activates MAPK1/ERK2, important mediators of the entire MAPK pathway. The overall effects include block of apoptosis, stress response and cell proliferation

B-RAF is immediately downstream of RAS and triggers MAP2K1/MEK, which activates MAPK1/ERK2, important mediators of the MAPK pathway. Then, the activating signal is passed on by a chain of kinase reactions, overall establishing MAPK signaling pathway [30]. Consequently, activating mutations of *KRAS* and *BRAF* produce at last the activation of this pathway, which is crucial for pancreatic cancer (Fig. 2). Interestingly, MAPK kinases are classified into three classes based on their distinctive effectors: extracellular signal-regulated kinases (ERK), Jun amino-terminal kinases (JNK) and *p38*-mitogen-activated protein kinases (p38MAPK) [37]. Each class regulates distinct functions: ERK is involved in proliferation, JNK in apoptosis and differentiation and p38MAPK in stress responses [37]. To enact specific cell responses, activated MAPK kinases translocate into the nucleus, where, through phosphorylation of different transcription factors, they can directly modulate the expression of different genes [30, 38]. Additional mechanisms that may replace *KRAS*/*BRAF* function for RTK/RAS/MAPK activation included mutations or amplifications of *GNAS*, *EGFR*, *ERBB2*, *MET*, *ERBB3* and *FGFR2* [9].

Regarding therapeutic approaches to MAPK pathway targeting, in addition to the above described MAPK inhibitors, some other interesting data come from a large, multicenter, non-randomized trial of 581 PDAC patients, as part of the so-called “Know Your Tumor” initiative [39]. In this cohort, wild-type *KRAS* tumors accounted for 8% of the whole PDAC cohort, and a significant proportion (24%) of these had alterations in other MAPK pathway effectors, including *BRAF* activating alterations. In this setting, patients who received molecular target therapy, including MAPK inhibitors and *BRAF*-targeted drugs, achieved an improved progression-free survival and median overall survival [38]. Other data regarding direct *BRAF* inhibitors come from a recent study, which reported a 6-months remission of a *BRAF*- mutant PDAC patient treated with the inhibitor dabrafenib [40]. Moreover, preclinical data indicate that even wt-*BRAF* might constitute a suitable therapeutic target in PDAC, taking advantage of the therapeutic synergism of “vertical” combination strategies simultaneously targeting BRAF and MEK along the same pathway, regardless of KRAS mutational status [41].

From the point of view of molecular pathology, an important consideration regards the specific *BRAF* mutation to be encountered in PDAC. Indeed, mutations that are the most common in other tumor types, including melanoma and thyroid carcinoma, are not frequently present in PDAC. Singhi and colleagues [11] reported typical *V600E* and *V600K BRAF* mutations in only about 1/5 of PDAC with *BRAF* alterations. This point requires the adoption of next-generation sequencing (NGS) technologies in clinical practice to guarantee in-depth analysis of the coding regions of *BRAF*, coupled with the possibility to investigate other *BRAF* alterations, such as the presence of fusion genes.

### Microsatellite instability (MSI) / defective mismatch repair (dMMR)

Another genetic alteration enriched in *KRAS* wild-type PDAC is microsatellite instability (MSI)/defective mismatch repair (dMMR) [11, 42–45]. A recent study has definitively demonstrated that MSI/dMMR PDAC harbor *KRAS* mutations less frequently than conventional PDAC, reaching statistically significant values [42]. However, it should be acknowledged that about one third of MSI/dMMR PDAC can present *KRAS* mutations [43, 44]. Microsatellites are short, repetitive DNA sequences of 1–6 base pairs present throughout the genome, mostly in noncoding regions [35]. Their repetitive nature renders them very sensitive to DNA mismatching errors, which can occur during DNA replication or iatrogenic damage [45]. Cancers harboring a dMMR are very often hypermutated and typically accumulate mutations in microsatellites: this condition is termed MSI [45].

**Fig. 3:**
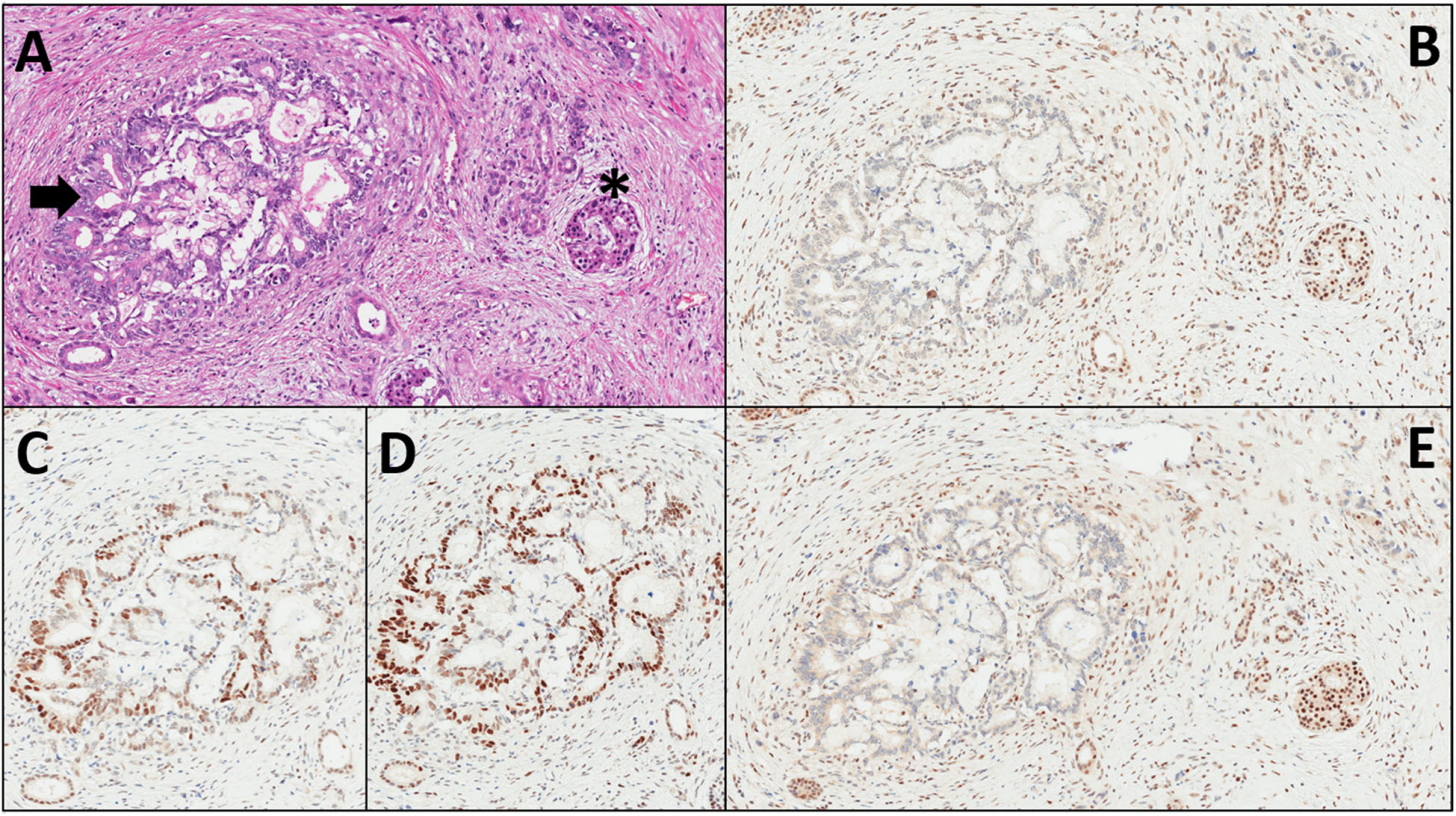
This is a classic example of a conventional pancreatic ductal adenocarcinoma (PDAC) with microsatellite instability, assessed by immunohistochemistry. **a** An infiltrating PDAC gland is centrally located on the left (arrow), and a normal endocrine islet is on the right (asterisk). Hematoxylin-Eosin staining, original magnification X10. **b** Loss of expression of the MMR protein MLH1 with immunohistochemistry. The infiltrating PDAC gland is totally negative (loss of protein expression), and the same time the endocrine cells of the islet are positive, as well as lymphocytes, endothelial and stromal cells in the periphery (the expression of the MMR proteins in non-neoplastic cells is used as an internal control demonstrating the reliability of the immunohistochemical analysis). Original magnification X10. **c**, **d** Conserved expression of the MMR proteins MSH2 (**c**) and MSH6 (**d**). Original magnification X10. **e** Loss of expression of the MMR protein PMS2 with immunohistochemistry. The infiltrating gland is totally negative (loss of expression), and the same time the endocrine cells of the islet as well as lymphocytes, endothelial and stromal cells in the periphery are positive. Original magnification X10

The MSI phenotype was first described in the familial cancer condition known as Lynch syndrome (LS), where the MMR genes *MLH1*, *MSH2*, *MSH6* or *PMS2* harbor germline mutations and portend marked susceptibility to develop several types of cancer, including PDAC [43]. MSI can be tested using immunohistochemistry (IHC) and molecular tests, including classic polymerase chain reaction (PCR) and next-generation sequencing (NGS) [45–47]. Immunohistochemical analysis is more reliable for cancers belonging to the spectrum of LS [43], where the loss of expression of the heterodimers MLH1-PMS2 and/or MSH2-MSH6 represent a very reliable surrogate of dMMR [44, 45]. For other cancer types, PCR or NGS approaches appear more robust for MSI/dMMR assessment and should be used instead of IHC. In PDAC, the use of IHC appears a reliable method (Fig. 3), although this cancer type does not represent one of the most common malignancies in LS patients [44, 45].

MSI in PDAC has been described with variable frequencies, with a prevalence ranging approximatively from 0.1 to 7% in the most recent investigations on large cohorts of patients [30, 48–51]. However, a recent comprehensive seminal paper has established its prevalence in PDAC at around 1–2% [44]. Highest values of MSI prevalence have been reached in specific PDAC subtypes, such as medullary variant, mucinous/colloid variant and IPMN-derived carcinomas [40, 44, 51]. Among conventional PDAC, Humphris et al., who interrogated 385 pancreatic cancer genomes for mutational signatures inferring defects in DNA repair, identified MSI in 1% of tumors [48]. Along this line, Hu et al., using an NGS assay designed to perform targeted deep sequencing of cancer-associated genes, found a 0.8% MSI frequency in a cohort of 833 PDAC [50]. Although it is a rare condition, the new frontiers of immunotherapy have opened new important therapeutic opportunities for tumors with this molecular alteration. Indeed, because of the defective mismatch repair machinery, tumors with MSI are hypermutated neoplasms; this genetic alteration leads to the synthesis of several (up to 50 times more than microsatellite stable tumors) aberrant and potentially immunogenic neo-antigens by the tumor cells [52]. An important self-response to the presence of these neo-antigens is a diffuse infiltration of the tumor area by cytotoxic T-cell lymphocytes (CTLs) [52]. Recent studies have highlighted the concomitant expression of multiple active immune checkpoint markers, such as the Programmed Cell Death Protein 1 (PD-1) and its ligand PD-L1. Their interaction cause T cells functional exhaustion and unresponsiveness [53]. Based on this discovery, Le et al. evaluated in 2015 the clinical activity of pembrolizumab, an anti-PD-1 immune checkpoint inhibitor, in a cohort of metastatic colorectal and non-colorectal carcinoma patients with or without MSI [54]. However, no patient with PDAC was included in this study. The results of this phase 2 trial clearly showed that MSI status was able to predict clinical benefits from immune checkpoint blockade therapy with pembrolizumab [55, 56]. In 2017, the same group published the “KEYNOTE-158” trial, investigating PD-1 blockade efficacy in patients with advanced dMMR cancers across different tumor types, including also PDAC [57]. If initially the objective response rate was similar between colorectal versus other tumor subtypes, an update of the trial including a total of 22 MSI/dMMR PDAC patients, showed only 4 of 22 patients with objective responses, which represented the lowest objective response among the different cancers investigated [58]. These findings highlight the complex biology of PDAC and call for further investigations in this field.

Of interest, tumor mutational burden (TMB) is another important variable in this setting, since it has been strictly correlated to the response to immunotherapy. TMB is a value that represents the rate of mutations in a tumor, and is expressed as the number of mutations per megabase. In PDAC, a high TMB is a rare finding and very often occurs simultaneously with MSI [11, 47, 50]. New NGS approaches can provide simultaneously data on MSI and TMB, with potentially immediate consequences on therapeutic opportunities for these specific PDAC molecular subgroups.

From the point of view of molecular pathology, it is important to acknowledge that IHC, performed using the antibodies for all 4 MMR proteins (MLH1, PMS2, MSH2 and MSH6) represents a very reliable methodology to assess MSI in PDAC. However, in cases of doubtful IHC results, MSI/dMMR should be assessed with PCR-based MSI or NGS. In cases with limited tissue (e.g.: endoscopic/ultrasound guided fine needle biopsy), NGS should be adopted as the first choice, as highlighted by a recent study on this topic [44]. Due to the potential importance associated with a diagnosis of MSI/dMMR in the context of PDAC, including eligibility for immunotherapy trials, it is of primary importance that all methodologies for MSI/dMMR assessment follow standardized protocols (including all pre-analytical phases) and up to date recommendations [45].

### Kinase fusion genes

The most important original report on this topic is a recent massive sequencing-based paper with a cohort of 3594 PDAC, where Singhi et al. showed that kinase fusion genes are one of the most frequent putative driver alterations in *KRAS* wild-type PDACs [11]. They found specific kinase fusions in *FGFR2* (12 cases), *RAF* (7 cases), *ALK* (5 cases), *RET* (4 cases), *MET* (2 cases), *NTRK1* (2 cases), *ERBB4* (1 case) and *FGFR3* (1 case), representing about 7.6% of genetic alterations of all *KRAS* wild-type PDAC. In the majority of fusion genes, a serine/threonine kinase or tyrosine kinase catalytic domain was fused to an oligomerization domain, which may represent a mutual mechanism of activation [11]. All these kinase fusions were mutually exclusive and, similar to *BRAF*, they were not present in PDACs with *KRAS* alterations [11].

Regarding the therapeutic approach to PDAC patients with kinase fusions, there are still limited data in the literature. Interestingly, a previous research identified 4 PDAC patients with *ALK* fusions; 3 of them showed stable disease with normalization of serum CA19-9 levels after treatment with an *ALK* inhibitor [59]. Notably, Pishvaian et al. recently described 2 PDAC patients with *NTRK1* fusions; such patients were already metastatic at the time of diagnosis but showed partial responses to the specific targeted drug entrectinib [60]. Regarding *BRAF* and *RAF* fusions, these molecular alterations have not been described in PDAC but in another exocrine pancreatic cancer, which is acinar cell carcinoma [61]. Intragenic/in-frame deletions in *BRAF* represent other potential driver alterations in *KRAS* wild-type PDAC. Although also in this case there are limited data, Aguirre et al. reported a single PDAC patient with oncogenic *BRAF* deletion with response to the pathway inhibitor trametinib [62].

In the last few years, several studies have detected neuregulin 1 (*NRG1*) fusion genes across different cancer types, particularly in invasive mucinous adenocarcinoma (IMA) of the lung and PDAC. Moreover, *NRG1* fusions have been detected with several fusion partners [63]. The *NRG1* gene encodes for a member of the epidermal growth factor (EGF) family, which binds to ERBB3, thereby causing its heterodimerization with ERBB2. *NRG1* gene fusions are generally in-frame and generate fusion proteins that maintain the extracellular EGF domain of NRG1 and the transmembrane domain of the rearrangement partner. Thus, the EGF domain of the fusion protein can constitutively bind to its partner and activate signaling through MAPK, PI3K-AKT, and NF-kB, increasing tumor proliferation and survival [63, 64]. In patients affected by PDAC, *NRG1* fusions are detected only in *KRAS* wild-type tumors and usually in the absence of other concomitant driver gene mutations, suggesting that the rare *NRG1* fusions act as an oncogenic driver in this tumor. In addition, some studies have demonstrated the actionability of *NRG1* fusions in PDAC. Indeed, when patients affected by heavily pretreated PDAC carrying *NRG1* fusion received matched therapy with afatinib, an irreversible ERBB1–4 inhibitor, clinical benefit and objective responses were documented [65, 66]. However, Drilon at al recently reported four patients affected by IMA of the lung with *NRG1* fusion that did not respond to afatinib, but showed an extraordinary clinical response to treatment with a new monoclonal antibody targeting ERBB3 [67].

From a molecular pathology perspective, the necessity to identify fusion genes further highlights the importance of introducing NGS into clinical practice. For PDAC patients, this point represents an urgent need, at least for *KRAS* wild-type PDAC. The final pathology report for PDAC should integrate morphology with molecular pathology, following the model of a next-generation histopathologic diagnosis [68].

## Clinical considerations and conclusions

Therapeutic management of pancreatic cancer remains an important clinical challenge and a clearly unmet medical need. With the notable exception of PDAC arising in the context of germline *BRCA1/2* mutations [69], no effective targeted therapies have been developed for this deadly disease and no routine molecular pathology testing is currently indicated at diagnosis. Even worse, in the past 20 years we have witnessed the failure of an awfully long list of clinical trials testing intriguing and pre-clinically sound molecularly targeted agents. Among many methodological faults that prevented the successful transition of many agents/combinations from early phase trials to phase III documentation of efficacy and registration [70], the most striking finding is that out of 37 trials involving biological agents, only one employed a biomarker-based population enrichment strategy. Testing targeted drugs in unselected PDAC populations has not met with clinical success, leading to a largely avoidable waste of time, resources and, most importantly, patients’ lives. In addition, it may have led to the dismissal of agents/strategies with potential efficacy in specific populations of patients. This is the case, using an example relevant to the topic discussed in this review, for the addition of trametinib (a potent allosteric MEK inhibitor) to gemcitabine as first-line treatment of advanced PDAC patients [13]: while ineffective in the entire (unselected) trial population, it might have benefitted the small (*n* = 40) population of *KRAS* wild type patients, in whom the risk of death was reduced by approximately 40% by the addition of trametinib, although with results that did not reach statistical significance [13].

As discussed here, *KRAS*-wild type PDAC may represent a distinct molecular subtype of pancreatic cancer. The genetic hallmarks in this category are represented by the presence of an activated MAPK, of MSI/dMMR, and of kinase-fusion genes in variable proportions. Differently from conventional PDAC, if appropriately selected based on their individual genomic and molecular features, these special PDAC subtypes can be treated with specific therapeutic strategies (see Table 1 for a list of selected ongoing trials with agents targeting molecular aberrations that are enriched in *KRAS* wild type PDAC), representing an important step towards the establishment of precision oncology for patients with pancreatic cancer [71]. As exemplified by the recently reported “Know Your Tumor” initiative experience in PDAC [72], only a minority of patients might currently benefit from extended molecular profiling (46 out of 1223–4% - profiled patients received matched therapy in their experience). That said, the evidences reviewed and discussed on these topics may call for a complementation of PDAC histological diagnosis with routine determination of *KRAS* mutational status, followed by comprehensive molecular profiling of *KRAS* wild-type cases.

**Table 1:** Selected trials targeting molecular aberrations enriched in *KRAS*-wt PDAC

Target	Tested drug	Phase trial	Population	Primary Outcomes
ALK	ceritinib	Phase I NCT02227940	Dose escalation (ALK negative) and expansion cohort (ALK positive) in advanced solid tumors in combination with standard chemotherapy Expansion cohort 2E: advanced pancreatic cancer ALK positive in combination with gemcitabine and nab-paclitaxel	MTD, RP2D
BRAF	binimetinib + encorafenib	Phase II NCT04390243	Pancreatic cancer BRAF mutated (V600E) after progression disease to at least one line of chemotherapy	ORR
MEK	cobimetinib or olaparib trametinib + hydroxychloroquine	Early Phase 1 NCT04005690 Phase I NCT03825289	Diagnosis of pancreatic cancer (resectable, borderline resectable, or advanced) are eligible Participants may be treatment naive or have received prior therapy Arm I: cobimetinib Arm II: Olaparib Pretreated Advanced Pancreatic Cancer	Assess the feasibility of collecting tumor tissue for biomarker evaluation prior to and after window therapy with either cobimetinib or olaparib DLT, RP2D
RET	selpercatinib sunitinib	EAP NCT03906331 Phase IV NCT02691793	Solid tumors RET activated RET fusion positive, FGFR2 fusion/FGFR mutation refractory solid tumor	- PFS
NTRK/ROS1	entrectinib	Phase II NCT02568267 (STARTRK2)	Solid tumors with NTRK1/2/3, ROS1, ALK gene rearrangments	ORR
NTRK	VDM-928	Phase I NCT03556228	Expansion phase: Solid tumors with NTRK1 gene fusions or amplification	AEs
NTRK/ROS1	DS-6051b	Phase I NCT02279433	advanced solid tumors harboring ROS1 or NTRK1, NTRK2, or NTRK3 rearrangement	DLT
NRG1	zenocutuzumab (MCLA-128)	Phase I/II NCT02912949	Dose escalation (NRG1 negative) and dose expansion (NRG1 positive) in advanced solid tumors Dose expansion Group G: pancreatic cancer with NRG1 fusion	AEs / SAEs ORR DOR Biomarkers analysis
HER2	A116	Phase I/II NCT03602079	Relapsed/Refractory Cancers Expressing HER2 Antigen or Having Amplified HER2 Gene	Phase I: MTD Phase II: ORR
	ACE1702	Phase I NCT0431975	Advanced or Metastatic HER2-expressing Solid Tumors	Safety, DLT, MTD, RP2D
	T-DXd	Phase2 NCT04482309	cohort six: patient with no satisfactory alternative treatment option affected by advanced pancreatic cancer with HER2 amplification	ORR
	afatinib + capecitabine	Phase I/IB NCT02451553	Phase I: pretreated solid tumor Phase IB: pretreated advanced pancreatic and biliary tract cancer	DLT, MTD, RP2D
ERK	LY3214996 +/− hydroxychloroquine	Phase II NCT04386057	Advanced pancreatic cancer	DCR
	ulixertinib/Palbociclib	Phase I NCT03454035	Dose escalation cohorts: histologically confirmed advanced refractory solid tumor	MTD
			Expansion: metastatic pancreatic cancer patients	OS
MSI	dostarlimab	Phase I NCT02715284	Part 2B: Cohort F non-endometrial dMMR/MSI-H or POLE-Mutated solid tumors, that have progressed following up to 2 prior lines of systemic for advanced disease	AE
FGFR	pemigatinib	Phase II NCT03822117	Cohort A Previously Treated Locally Advanced/Metastatic or Surgically Unresectable Solid Tumor Malignancies Harboring Activating FGFR 1–3 fusion	ORR
	infigratinib	Phase II NCT04233567	Cohort 1–2: solid tumor harboring FGFR1–3 fusion/translocation who have progressed on or are intolerant to standard of care	ORR
	erdafitinib	Phase II NCT04083976	Pretreated advanced solid tumor malignancy FGFR mutation or gene fusion	ORR
	debio 1347	Phase II NCT03834220	Pretreated Solid Tumors Harboring a Fusion of FGFR1, FGFR2 or FGFR3	ORR

*MTD* maximum tolerated dose, *RPD2* recommended phase II dose, *ORR* objective response rate, *DLT* dose-limiting toxicity, *PFS* progression free survival, *AEs* adverse events, *SAEs* serious adverse events, *DOR* duration of response, *OS* overall survival

## Data Availability

All data presented in this review are totally available and present in the text.

## Abbreviations

PDAC: pancreatic ductal adenocarcinoma
MAPK: mitogen-activated protein kinase
ERK: extracellular signal-regulated kinase
IMA: invasive mucinous adenocarcinoma
JNK: Jun amino-terminal kinase
p38MAPK: p38-mitogen-activated protein kinase
MSI: microsatellite instability
dMMR: defective mismatch repair
MMR: mismatch repair
LS: Lynch syndrome
IHC: immunohistochemistry
PCR: polymerase chain reaction
NGS: next-generation sequencing
CTLs: cytotoxic T-cell lymphocytes
PD-1: programmed cell death protein 1
PD-L1: programmed cell death protein 1 – ligand
TMB: tumor mutational burden
